# Immersive Virtual Reality Field Trips Facilitate Learning About Climate Change

**DOI:** 10.3389/fpsyg.2018.02364

**Published:** 2018-11-30

**Authors:** David M. Markowitz, Rob Laha, Brian P. Perone, Roy D. Pea, Jeremy N. Bailenson

**Affiliations:** ^1^School of Journalism and Communication, University of Oregon, Eugene, OR, United States; ^2^Department of Neurobiology, Stanford University, Stanford, CA, United States; ^3^Department of Learning Sciences and Education, Stanford University, Stanford, CA, United States; ^4^Department of Communication, Stanford University, Stanford, CA, United States

**Keywords:** immersive virtual reality, climate change education, ocean acidification, learning, education

## Abstract

Across four studies, two controlled lab experiments and two field studies, we tested the efficacy of immersive Virtual Reality (VR) as an education medium for teaching the consequences of climate change, particularly ocean acidification. Over 270 participants from four different learning settings experienced an immersive underwater world designed to show the process and effects of rising sea water acidity. In all of our investigations, after experiencing immersive VR people demonstrated knowledge gains or inquisitiveness about climate science and in some cases, displayed more positive attitudes toward the environment after comparing pre- and post-test assessments. The analyses also revealed a potential *post-hoc* mechanism for the learning effects, as the more that people explored the spatial learning environment, the more they demonstrated a change in knowledge about ocean acidification. This work is unique by showing distinct learning gains or an interest in learning across a variety of participants (high school, college students, adults), measures (learning gain scores, tracking data about movement in the virtual world, qualitative responses from classroom teachers), and content (multiple versions varying in length and content about climate change were tested). Our findings explicate the opportunity to use immersive VR for environmental education and to drive information-seeking about important social issues such as climate change.

## Immersive virtual reality field trips facilitate learning about climate change

Virtual experiences can be psychologically impactful. Decades of research on Virtual Reality (VR) suggest that people internalize their virtual experiences and treat them as real (Blascovich and Bailenson, 2011). For example, people who have high anxiety about public speaking and give a speech in VR respond to feedback they receive from a virtual audience. If the audience appears negative, people become anxious and internalize reactions from the virtual world (Pertaub et al., 2002). More recent scholarship suggests that the activities in virtual spaces also provide a window into social and psychological processes. That is, the physical characteristics of an avatar can affect interpersonal dynamics (Yee and Bailenson, 2007) and embodying someone of a minority race (e.g., a black avatar) reduces levels of bias toward black individuals based on their skin color (Peck et al., 2013).

The prior evidence suggests that VR can be used to evaluate experiences that are both common (e.g., physical characteristics can affect social dynamics) and fantastical (e.g., changing one's race) relative to everyday life. An opportunity in the VR literature, due to an underwhelming number of experimental studies and mixed evidence, is the evaluation of *immersive* VR as a medium for education. The word immersive is italicized because there are numerous studies evaluating desktop VR as a medium for learning (see Dede, 2009; Merchant et al., 2014), but there are a surprisingly small number of quantitative studies examining immersive VR as a tool for learning.

The connection between immersive VR and education has likely been underdeveloped because there are challenges associated with using virtual technology to facilitate learning. According to early but still relevant work by Bricken (1991), cost (e.g., purchasing your own VR system is expensive), usability (e.g., the technology or experiences in VR can be unintuitive), and fear (e.g., being placed in a fully immersive space can be daunting) are three constraints for learning in virtual environments. The novelty of a virtual experience may undermine VR's effectiveness as an educational tool and most people do not currently own immersive VR hardware—or have limited experience with the technology. Learning about science, for example, during a first experience in VR may therefore be impacted by the medium's unique infrastructure. The integrity of the virtual world must also meet the expectations of an individual if he or she could perform the task in the physical world. That is, practicing surgery in VR must replicate a genuine, unmediated surgical experience including accurate hand motions, sounds, and biological representations.

The current paper creates learning content and implements the idea of taking virtual field trips to begin to address these challenges. Specifically, we test whether people can learn about the consequences of climate change in immersive VR. Our interest in climate change education stems from the importance of bringing complex issues closer to people psychologically (Schuldt et al., 2016) and is also inspired by recent calls for innovative teaching methods to address environmental concerns (e.g., mobile games; McCright et al., 2013; Wu and Lee, 2015). In this spirit, we present two laboratory experiments and two field studies that demonstrate how an immersive VR experience can facilitate learning or an interest in learning about climate change.

## A primer on virtual reality

VR is a communication medium that leads an individual to perceive experiences and environments as if they were not synthetic (Lombard and Ditton, 1997). A virtual experience hinges on two critical dimensions: immersion and presence. Immersion considers how well the technology approximates actions and movements in the virtual space. For example, a highly immersive experience tracks a person's arms or legs with high levels of accuracy and maps his or her movements to the virtual space in a natural way. Presence, on the other hand, is the psychological sense of “being there” (Heeter, 1992; Slater and Wilbur, 1997). Presence reflects how well an individual perceives the self in the virtual world and has the opportunity to act within the space (Wirth et al., 2007). Together, an immersive virtual experience with high levels of presence allows the individual to suspend any belief that the experience is mediated.

VR disciplines have a rich history of documenting the medium's role as an important tool to evaluate social and psychological phenomena. For example, VR assists medical fields with patient rehabilitation and psychological interventions (Nilsson et al., 2012). That is, military veterans who relive their Vietnam War experiences in VR show reduced levels of Post-Traumatic Stress Disorder relative to baseline, nearly 6 months after exposure (Rothbaum et al., 2001). People who experience VR therapy for anxiety or personality disorders (e.g., Wiederhold and Wiederhold, 2005; Nararro-Haro et al., 2016), social phobias (e.g., Klinger et al., 2005), and other fears (e.g., spiders, flying, heights) show consistent or enhanced levels of psychological improvement relative to traditional, face-to-face treatments. Medicine, as well as other fields (e.g., athletics; Bideau et al., 2010) have also used virtual technology for teaching and training because repetitions in VR are free. It is often financially and ethically expensive for doctors, trainers, or nurses to practice on humans, but VR offers rehearsals that feel real and are relatively low-cost. Cadavers can cost hospitals thousands of dollars and harming a living person has non-trivial consequences, including in extreme cases, death. VR allows doctors to move within a space that imitates their physical environment with carryover effects that can positively impact operating room performance (Seymour et al., 2002).

### Virtual reality and science education

Since the 1990's VR has been tested and used as a tool for Science, Technology, Engineering, and Mathematics (STEM) education. According to Salzman et al. (1999), learning in VR is possible because VR systems produce highly engaging experiences, which can lead to greater focus on the learning topic (Bricken and Byrne, 1993). VR puts users within a virtual learning environment that reflects the topic of interest (e.g., placing students within a cell if they are learning about human biology), which can offer perspectives that are difficult to realize in other learning settings (see Johnson et al., 2002). VR can be effective for learning because it enables “users to interact with spatial representations” from many frames of reference (Salzman et al., 1999, p. 294). Finally, the addition of auditory, haptic, and other sensory feedback allows users participate in a world that feels unmediated. Such features provide a multidimensional experience that can aid in cognitive processing and information retention (Ragan et al., 2012).

A classic example of VR developed for learning about science was created by Dede (2009), who designed a desktop VR program called River City (see Clarke and Dede, 2009). The program's goal is to teach students about the scientific method by progressing through different parts of the city. Students develop research questions about why River City is experiencing health issues or why people are getting sick at uneven rates. Participants move through the curriculum by collecting, analyzing, and interpreting science data.

An extensive review of the research on educational virtual environments by Mikropoulos and Natsis (2011) suggests that most VR programs are consistent with River City, using desktop or non-immersive technology for learning (e.g., computers, styluses, joysticks). For example, Minogue et al. (2006) gave middle school students a desktop VR program to explore the details of a cell. In the treatment group, students received visual and haptic feedback as they explored a virtual cell and the control group only received visual feedback. The data revealed positive learning effects for students in both groups after comparing pre- to post-test knowledge scores about cells. Haptic feedback improved knowledge scores for some low-achieving students, but overall, it did not lead to learning differences between groups.

The prior patterns have been summarized at scale in a recent meta-analysis that evaluated the effect of desktop VR instruction on learning and knowledge gain (Merchant et al., 2014). Across nearly 30 studies, Merchant et al. (2014) observed that desktop VR interventions produce a significant, positive effect on leaning outcomes and knowledge gain in K-12 higher education. We use this meta-analytic evidence as support for the idea that instruction through predominantly desktop VR is effective and can lead to positive knowledge gain for many science topics. It is still unclear, however, if immersive VR can facilitate learning about complex social and environmental issues.

## The affordances of immersive VR for learning

An experience in immersive VR should lead to positive learning effects for several reasons. From a technical perspective, immersive VR hardware creates a sensory experience that surrounds a user. Immersive VR is characterized by the use of a Head Mounted Display (HMD), enhanced with auditory, haptic, or other sensory feedback to give the virtual world a genuine look and feel (Ahn et al., 2016). Therefore, the virtual world is perceived as unmediated and people often respond to stimuli as they would outside of a virtual world. Immersive VR also affords situated action, or the ability for people to move and act within a novel setting. Situated action can facilitate knowledge gain because learning occurs in the environment of interest (e.g., learning about ocean acidification occurs while placed virtually underwater) and people can actively engage with the environment (e.g., move arms or legs in the virtual space; Gallagher, 2005). This idea is rooted in embodied cognition research (Clark, 1997), which generally suggests that people can develop strong social and psychological attachments to an environment by interacting and moving within it. That is, people can learn, form more positive associations toward a stimulus, and internalize information by acting in a space and performing motions that are relevant to a specific context relative to simply internalizing information about a phenomenon (Weisberg and Newcombe, 2017). This rationale is also supported by media interactivity theories such as the Interactive Information Processing Model (Tremayne and Dunwoody, 2001). This framework suggests that interactivity with media, elaborative cognitive processing, and information recall are positively associated, which can also extend to virtual worlds as well. Therefore, interacting with an immersive virtual environment can lead to deeper cognitive processing and data retention for complex information such as ocean acidification.

### Studies demonstrating that immersion facilitates learning

Learning about science in immersive VR has been an understudied phenomenon and to our knowledge, few quantitative studies have evaluated the effect of using immersive VR in classrooms to teach about impending environment issues such as climate change. One study by Vishwanath et al. (2017) integrated Google Cardboard and Google Expeditions into classrooms in India and qualitatively evaluated student engagement in the classroom relative to when VR was not present (classes before VR was introduced). The authors reported that classes before VR were superficial and dull, compared to VR-augmented classes that were engaging and interesting. For example, Vishwanath et al. (2017) highlight that the number of questions asked in class and the length of discussions increased during the VR classes relative to the non-VR classes.

In a different laboratory study, participants were randomly assigned to one of three VR systems (e.g., a system similar to the CAVE, a HMD system with tracking, a HMD system without tracking) and or a non-VR control system to learn about four topics: astronomy, transportation, networking, and inventors (Alhalabi, 2016). Learning was assessed by quizzes on these topics and the results suggest that any form of VR leads to more positive learning outcomes relative to no VR. Another study evaluated if the cognitive modifiability of young children, defined as the capacity to adapt and adjust to complexity, was affected by the medium that they learned how to recall the orientation, position, and other characteristics of blocks (Passig et al., 2016). Children who were assigned to an immersive VR condition, relative to desktop VR, tangible blocks, or control conditions, demonstrated greater cognitive modifiability, which is evidence of enhanced analytic thinking. Finally, prior work also suggests that an experience in immersive VR can lead to more positive learning gains relative to lecture-based instruction for United States soldiers, who were educated on military procedures pertaining to corrosion prevention and control (Webster, 2016).

### Studies demonstrating that immersion inhibits learning

Other studies have found mixed effects for the impact of immersive VR on learning. For example, recent work suggests that students report greater presence in immersive VR (with a HMD) relative to desktop VR after a biology simulation, but they learn less in immersive VR (Makransky et al., 2017). Similar reduced learning effects in immersive VR relative to desktop VR have been found after comparing learning scores for students who learn biology (immersive VR vs. a PowerPoint presentation; Parong and Mayer, 2018). Finally, equivalent levels of learning have also been noted between immersive VR and desktop VR in other science topics (Moreno and Mayer, 2002). Students learned about botany and experienced a tutorial on how to design a plant that could live in low sunlight. Participants in the HMD condition perceived the experience as more immersive relative to the desktop computer condition, but information retention was the same across media.

With mixed effects of immersive VR on learning about science, new studies are needed to add to this body of evidence that can also test the boundary conditions for immersive learning. Here, we evaluate if immersive VR is an effective medium to generate positive learning outcomes and perform exploratory studies to investigate if other parts of a virtual experience (e.g., embodiment, contextual movements) can affect learning about climate change and the environment.

## Environmental education

In general, environmental education is difficult because the issues are complex and challenging to internalize. Prior research suggests that people may feel undecided or uncertain about climate change, specifically, because they cannot see the environment changing first-hand or on a regular basis (Schuldt et al., 2016). The time lag between negative actions (e.g., pollution) that lead to environmental changes may motivate people to believe that a single phenomenon cannot cause such extreme outcomes (Weber, 2013). These ideas suggest that if environmental issues were perceived as psychologically closer or brought to observers directly (Pearson et al., 2016), perhaps people could learn about the issues and be encouraged to take pro-environmental action.

Recent work has proposed that to address the challenges associated with climate change education, innovative teaching methods are needed for public engagement on the complex social and environmental issues. Wu and Lee (2015) suggest that mediated experiences, particularly involving games about climate change, allow people to dynamically learn through “doing and being” rather than perceiving information in a lecture format (p. 413). Teaching science in a mediated manner provides first-hand experiences and can trigger an emotional response in a user compared to simple information exposure (Mendler de Suarez et al., 2012). Therefore, according to recent scholarship, some of the concerns associated with VR for learning are lifted because of improved technology (e.g., people can play mobile games to interact with a virtual world outside of the lab) and an increased focus on eliciting psychological attachment to the issues (Wu and Lee, 2015).

We propose that learning about the consequences of climate change should occur in immersive VR because participants will experience negative environmental events first-hand and the issues will be made psychologically closer. These ideas are also supported by theories of psychological distance, such as Construal Level Theory, which suggests that experiences are often appraised based on how spatially, temporally, or socially proximate (or distant) they are to an individual (Trope and Liberman, 2010). If an object or event is perceived as psychologically proximate, it is often internalized in concrete terms, compared to an event that is perceived as psychologically distant and internalized in abstract terms (Margolin and Markowitz, 2018). These construal patterns have downstream effects; for example, people who construe objects in concrete terms tend to be less risky than people who construe objects in abstract terms because the effects of their decisions are viewed as more immediate (see Raue et al., 2015 for a review). Therefore, data suggest that a detailed and concrete thinking style can bring a social issue psychologically closer, which then encourages people to appraise and feel connected to information. For a pressing and complex issue such as climate change, construing ocean acidification as a psychologically proximate phenomenon through immersive VR and having people internalize the effects of problematic environmental behavior, is a worthwhile and theoretically grounded effort.

Our rationale is also consistent with Dede (2009), who suggests that immersive technology can enhance education “by allowing multiple perspectives, situating learning, and transfer” (p. 66). We use the opportunities afforded by immersive VR, such as bringing an abstract event closer and situating the learning experience, to test the idea that people can demonstrate knowledge gain about the consequences of climate change. Specifically, we address a phenomenon called ocean acidification, or the process of the ocean's water decreasing in pH due to increasing carbon dioxide in the atmosphere.

### Ocean acidification

Ocean acidification is the decreasing pH of the world's surface water as a result of increasing atmospheric carbon dioxide levels, primarily due to human combustion of fossil fuels (Doney et al., 2009). This process has been well documented in fieldwork and the effects of ocean acidification are particularly noticeable on calcifying organisms, as the carbonic acid formed by the carbon dioxide both breaks down and prevents the formation of their exoskeletons (Hoegh-Guldberg et al., 2007). Ocean acidification has precipitous consequences as well. Changes to calcifying organisms affect the competitive interactions of species farther up the food chain (Kroeker et al., 2012). Scientists agree that the rate of acidification is expected to rise in the future, with even more dramatic effects on ocean ecosystems and other entities that rely on them (Doney et al., 2009).

To understand the consequences of ocean acidification, scientists have observed naturally occurring sources of sub-oceanic carbon dioxide produced by underwater volcanic vents, for example one site near the coast of Ischia, Italy. The underwater processes in this region provide scientists with a “crystal ball” to see what will happen to oceans as more carbon dioxide is added to the atmosphere (Hall-Spencer et al., 2008; Kroeker et al., 2011). Despite the severity of the problem and the well-supported research behind it, ocean acidification knowledge remains low and the phenomenon receives far less public attention than other climate change issues in environmental education (Kroeker et al., 2012; Capstick et al., 2016). The natural case study of ocean acidification in Ischia Italy, paired with recent calls for innovative methods that emphasize behavior and attitude change to improve environmental education (McCright et al., 2013; Wu and Lee, 2015), motivated our experiments to test if people can learn about climate change consequences in immersive VR.

We investigated the effectiveness of immersive VR for learning about marine science through virtual field trips, a common metaphor for learning experiences in a virtual world (Borst et al., 2018). Our first study started at a high school in the West Coast of the United States. In immersive VR, students participated in activities based on traditional practices of marine scientists, who dive underwater and interact with species of flora and fauna to measure biodiversity. The underwater virtual world was modeled after the Ischian Reef in Italy, where naturally occurring carbon dioxide vents acidify certain areas of the reef and provide scientists with a window into a future affected by climate change. We were primarily interested in the effect of immersive VR—characterized by haptic, auditory, and other sensory feedback experienced throughout a virtual world (Ahn et al., 2016)—on the students' learning and knowledge gain about ocean acidification. Our subsequent studies were exploratory and tested the effect of embodiment (Study 2), movement (Study 3), and situated action (Study 4) on learning gains or an interest in learning about climate change.

## Study 1

Our study began with a collaboration that involved two high school science teachers, who co-organized an immersive VR experience that allowed students to experience the effects of ocean acidification in their own classroom. This VR experience was originally developed based on work by prior researchers and adapted for our research design (Kroeker et al., 2011, 2012). Over 6 months, teachers and researchers met to develop the VR experience in an iterative process, involving multiple revisions of both the VR experience and the learning measures. We collaboratively identified four main design goals: (1) *physical immersion*, or making the virtual world of the ocean ecosystem appear to visually surround the learner in space, (2) *embodiment*, where students should have the sense of feeling present in a virtual body connected to the ecosystem, (3) *natural interactions*, or the idea that the students should interact with the underwater world in a manner that felt unmediated, and (4) *time travel*, or the ability for students to see the accelerated effects of ocean acidification on the world around them.

We also identified two main learning goals for the immersive VR experience: (1) demonstrating knowledge gain about the process and causes of ocean acidification, and (2) changing attitudes toward the ocean environment and ocean acidification.

## Method

### Participants

The collaborating school teachers had substantial teaching experience (*M* = 23 y) and taught this particular marine biology class for 2 and 4 years, respectively. A total of 19 students (7 female) between the ages of 16 and 18 (*Mdn* = 17) volunteered to participate in our research project. For the final delayed post-test, two were absent and one student chose not to participate. No compensation was given for participation in this research project.

### Materials

The immersive environment was created with Worldviz's Vizard software, and rendered by a Dell Precision T7500 running Windows 7 with 12 GB of memory and a NVIDIA GTX 680 graphics card with 1.5 GB of video memory. Participants in the VR experience viewed the world through an Oculus Rift DK2 HMD, providing three-dimensional stereoscopic views at a resolution of 960 × 1,080 pixels per eye at a refresh rate of 75 Hz (e.g., 75 frames per second, per eye) and 30 ms latency. The HMD includes an internal sensor that tracks orientation of a user's head at a rate of 1 kHz. The external Oculus Rift infrared sensor was used to track participants' position in the virtual space, providing six degrees-of-freedom of head tracking, even though participants did not physically walk around. The virtual world was visually complex, consisting of ~400,000 polygons depending on the participant's visual field, with animated fish and sea life throughout the experience.

Participants stood throughout the virtual experience, hand-pressed the buttons of a standard mouse to interact with the virtual world, and wore stereo headphones. In the classroom, two separate computer and HMD systems were used simultaneously in different parts of the room.

### Procedure

**Figure 1:**
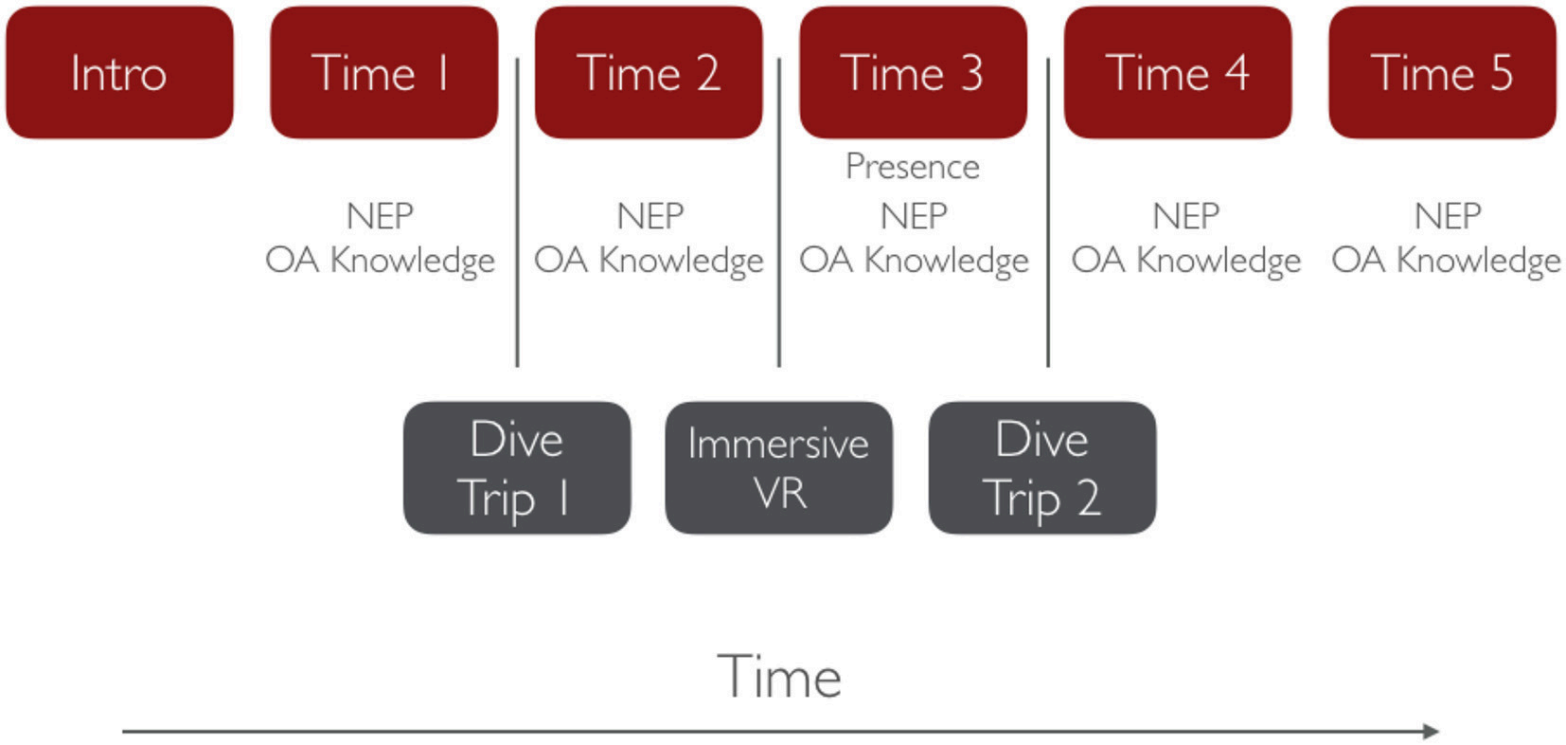
Procedure for Study 1. The measures recruited from each participant are represented under each stage of the study. OA, Ocean Acidification; NEP, New Ecological Paradigm.

The experiment consisted of six classroom meetings with students and their teachers. All parts of the current study occurred before the topic of ocean acidification was covered in the students' coursework. All activities were approved by our university's Institutional Review Board and sanctioned by the teachers. An overview of the procedure is provided in Figure 1.

In the first introductory meeting, students signed up for the study and participated in a VR demonstration unrelated to ocean acidification or science. This allowed the hardware to be tested outside the lab and ensured that the ocean acidification experience was not the students' first VR exposure, resolving one of Bricken's (1991) concerns about the potential fear associated with a first virtual experience. Two weeks later, the research team administered a questionnaire to obtain students' baseline knowledge of ocean acidification. The questionnaire contained inquiries into ocean acidification and the New Ecological Paradigm (NEP), a scale to measure attitudes toward the environment. The following day, all students in the class, which included four subjects from our study, went on the first of two open-water scuba diving trips in physical space. This first underwater trip is henceforth referred to as Dive Trip 1 and was introduced to evaluate the potential impact of a physical-world experience on a virtual-world experience. The following week, the research team re-administered the pre-test materials to all participants.

**Figure 2:**
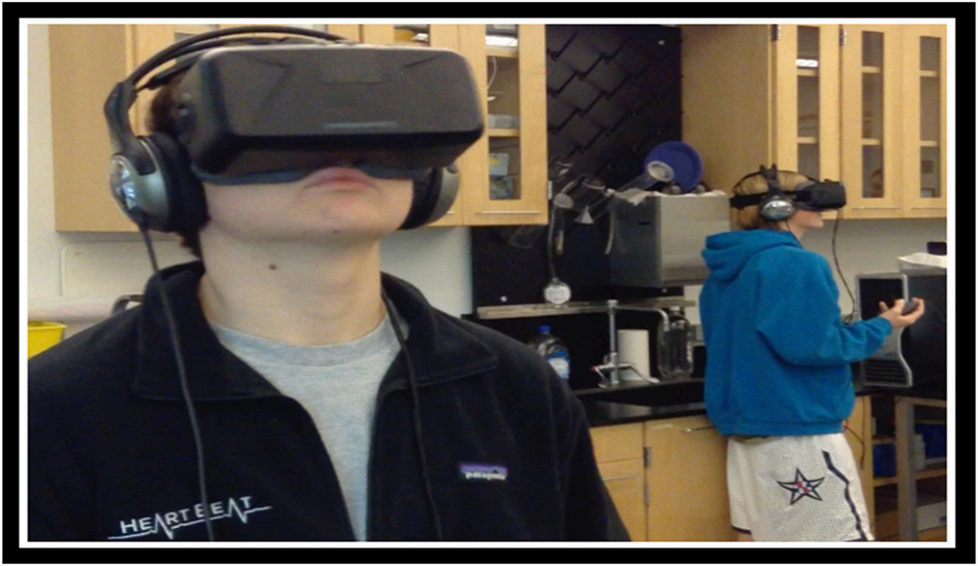
This photo was taken during an actual VR learning session at a private high school. The school students pictured, and their parents, signed a release for their photos to be used in association with this study.

One month after the pre-test, the research team returned to the classroom with the immersive VR hardware. Students were randomly assigned to one of two immersive VR stations and wore a HMD and headphones (Figure 2), but were not tested for stereo vision. Other students who were not participating at the time were given unrelated assignments to complete at their desks.

**Figure 3:**
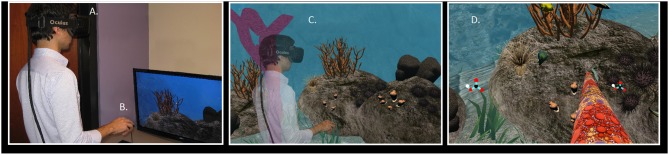
**(A)** The participant wears an Oculus Development Kit 2 (DK2) VR display. **(B)** The participant holds a mouse in-hand, using the left mouse button to interact with objects in the virtual environment. **(C)** The participant embodies a piece of coral on the virtual rocky reef. When the participant looks down at their virtual body, they see the purple coral stock. When the participant looks to the left and right, they see their purple coral arms. **(D)** The participant attempts to collect calcium bicarbonate ions. By clicking the left mouse button, the participant can extend their coral polyp arm out in front of them in order to collect the ions that float by in the water. Note, the external Rift infrared sensor was used, providing six degrees-of-freedom of tracking.

In the immersive VR session, a synchronized narration told participants that their body had become a coral (Figure 3). They were also introduced to the sea life and heard about the causes, processes, and effects of ocean acidification. Participant head rotation was tracked via the HMD and their position in X, Y, Z space was tracked via the infrared sensor. They were also told that their coral avatars would be fixed in the VR experience and were told to refrain from walking around in the physical room. Participants controlled their coral arm movements with the mouse and collected a variety of objects, including calcium and bicarbonate ions to stay healthy as a coral.

As this process continued, subjects viewed the ill-effects of ocean acidification on the surrounding ecosystem over the next 40 years (e.g., a decrease in fish populations, increased algae levels). Gradually, the underwater scene morphed into how marine scientists expect it will look at the end of this century and participants were instructed to observe how the acidity dissolved their own coral body. The immersive VR experience concludes with the reef returning to its original state and the participant hears that there is still time left to salvage what we have.

After the VR experience, students completed a questionnaire at their desks, which included the six questions about ocean acidification, the NEP scale, and a self-reported measure of presence. The following day (e.g., after the immersive VR classroom experience), the class held their second open water scuba dive, henceforth called Dive Trip 2, which included 10 participants from our study. The next week, the research team administered the ocean acidification knowledge questionnaire and the NEP questionnaire. Four weeks after the VR experience, the research team again administered a delayed post-test. Two students were absent and one chose not to participate in this post-test, yielding 16 respondents for the final measurement, only.

### Measures

Scale and inter-rater reliability statistics for all studies are represented in Table 1 and all measures met acceptable to exceptional levels of internal consistency or agreement.

**Table 1:** Scale and inter-rater reliability statistics across studies.

**Study (location)**	**OAK**	**NEP**	**CNS**	**Presence**	**Simulator sickness**
Study 1 (High school)	0.97	0.88	—	0.96	—
Study 2 (Laboratory)	0.98	0.83	0.86	0.78	—
Study 3 (Tribeca Film Festival)	—	—	—	—	—
Study 4 (Laboratory)	0.52	0.86	0.81	0.88	0.65

*OAK, Ocean Acidification Knowledge; NEP, New Ecological Paradigm; CNS, Connectedness to Nature Scale. All scale reliability analyses used Cronbach's alpha. Agreements on OAK ratings used Cohen's kappa, except Study 4 which used Krippendorff's alpha*.

#### Ocean acidification knowledge

To assess if learning about ocean acidification occurred in immersive VR, students submitted responses to six open-response questions: (1) What is ocean acidification?, (2) What is the most significant cause of ocean acidification?, (3) What are some of the predicted effects of increasing ocean acidification on the world's ocean ecosystems? Be as detailed as you can., (4) How do coral and other sea creatures build their hard skeletons and shells?, (5) How much carbon dioxide is absorbed by the planet's oceans each year?, and (6) How much has the pH level of the ocean changed since the industrial revolution?

All questions were scored independently by two coders, who were instructed to grade the responses based on accuracy. Consistent with prior work (Minogue et al., 2006), responses were ranked on a point system: zero points for no answer or simply restating the question, one point for a partially correct answer, and two points if the answer contained nearly perfect accuracy with minor inaccuracies. As expected, average ocean acidification knowledge during the pre-test time blocks (Time 1 and 2 in Table 2) was low, [(*M* = 1.97, *SD* = 1.43); min = 0, max = 5.0], compared to the average ocean acidification knowledge during the final three time blocks, [(*M* = 4.62, *SD* = 2.52); min = 0.5, max = 10.5]. Inter-rater reliability for these judgments was high (Table 1) and examples of ideal answers are provided in the Appendix A. Note, because there were six questions, knowledge scores could have reached a maximum of twelve points.

**Table 2:** Ocean acidification knowledge over time for study 1.

**Time**	***M***	***SE***	**95% CI**
1	1.97	0.50	[0.97, 2.98]
2	1.97	0.50	[0.97, 2.98]
3	5.08	0.50	[4.08, 6.08]
4	4.47	0.50	[3.47, 5.48]
5	4.42	0.52	[3.37, 5.47]

*Time corresponds to the blocks represented in Figure 1*.

#### Environmental attitudes

The attitude questionnaire included items from the New Ecological Paradigm (NEP; Dunlap et al., 2000), on seven-point Likert scales, ranging from “strongly disagree” to “strongly agree.” Participants responded to assertions such as “We are approaching the limit of the number of people the earth can support,” “Humans were meant to rule over the rest of nature,” and “The balance of nature is very delicate and easily upset.” Responses to these items were highly correlated (Table 1). We averaged participants' responses to create an overall measure of environmental concern, with higher values indicating greater concern levels than lower values, [(*M* = 4.77, *SD* = 0.84); min = 2.8, max = 6.8].

#### Presence

To measure participants' sense of presence, we adapted a 10-item scale from prior immersive VR research (Yee and Bailenson, 2007). All questions were seven-point Likert scales, ranging from “strongly disagree” to “strongly agree.” Participants were asked to rate their agreement with statements such as, “I felt surrounded by the virtual world,” “I felt like I was on a reef,” and “The decay of the virtual world around me affected me personally.” The responses to these items were averaged, [(*M* = 5.21, *SD* = 1.36); min = 1.1, max = 7.0].

## Results and discussion

The data were analyzed using hierarchical linear mixed models with a random effect for participant to account for multiple, non-independent observations by each student. Note, data and experimental scripts are available from the authors upon request.

### Ocean acidification knowledge

As expected, there was a significant effect of time on knowledge about ocean acidification, [*F*_(4, 69.08)_ = 18.85, *p* < 0.001, η^2^ = 0.50]. Table 2 displays the trend of knowledge gain over time and a crucial increase in ocean acidification knowledge after the immersive VR intervention (from Time 2 to Time 3). *Bonferroni*-corrected adjustments support this pattern as ocean acidification knowledge was significantly higher for all time points from Time 3 onward, compared to Times 1 and 2 [*p*'s < 0.001].

Considering students who participated in at least one of the open-water dives during the study's time period, we observed a significant Dive Trip (trip 1, trip 2) X Time (first, second, third, fourth, fifth meeting) interaction, *F*_(4, 46.21)_ = 2.85, *p* = 0.034. Further inspection of this pattern revealed that only during the last session (Time 5), students who went on the first open-water dive reported significantly more ocean acidification knowledge than participants who went on the second dive (*p* = 0.009). This suggests that a physical-world experience may have a positive impact on learning about complex marine phenomena if it precedes the virtual world. The effect is downstream and not immediate, however. We interpret these data with caution because this study was not a controlled experiment and there may be other factors accounting for the difference between the diving conditions.

### Environmental attitude

There was no effect of environmental attitude over time or between dive trips [*p*'s > 0.15, η^2^ < 0.01].

### Presence

There was no difference in reported levels of presence for participants who went scuba diving before (*M* = 4.05, *SD* = 2.03) or after (*M* = 5.87, *SD* = 0.77) the immersive VR experience, [Welch's *t*_(3.35)_ = −1.74, *p* = 0.17, *d* = 1.18].

This study provides evidence that students in a high school classroom setting can learn about marine science in immersive VR. As expected, overall student knowledge about ocean acidification was low at the beginning of the program. The relatively stable knowledge gain, weeks after the immersive VR field trip, demonstrates the potential of using this technology to facilitate learning over time. An initial open-water scuba dive seems to have increased the effectiveness of the virtual experience, while the exact mechanism remains unclear and our relatively small sample prevents broad conclusions to be made from these data. Finally, we observed a null effect for changes in environmental attitudes and presence. These patterns may be genuine or perhaps an artifact of an underpowered design. We therefore continue to explore these dimensions in future studies.

We also report that no pragmatic issues occurred when deploying an immersive VR experience outside of the lab. Students did not get sick, nor did they find the immersive VR experience disruptive in their learning experience. These outcomes were not empirical certainties and should not be overlooked.

There are important limitations to this experiment that we address in Study 2. First, we transition the experimental design to a controlled lab setting that allows for a tighter manipulation of the immersive VR experience. Our high school sample was also admittedly small. In Study 2, we recruit a larger number of university undergraduates to participate in our experiment and perform an exploratory study to investigate if different avatar embodiment may affect knowledge gain and how people feel about ocean acidification. Embodiment is often an important method to induce attitude change and behavior (Kilteni et al., 2012), but has seldom been applied to learning about climate change. We therefore position this study as exploratory to investigate how different avatar embodiment affects learning.

## Study 2

The materials in Study 2 were similar to Study 1, with minor virtual world modifications. Most significantly, two conditions were created, each with a different self-avatar for participants to embody. Half of the participants saw themselves as a coral in the virtual world (consistent with Study 1), while others remained human and saw themselves as scuba divers on the seafloor.

If the power of an immersive VR experience comes from a sense of connectedness to one's avatar, then becoming someone or something more centrally connected to the virtual world (e.g., the underwater reef) should result in greater learning gains and greater environmental concerns. We expect knowledge gains from pre- to post-test assessments for participants across conditions and explore if embodying a coral or a scuba diver affects learning and attitude change.

## Method

### Participants

Forty-seven undergraduate and graduate students from a large West Coast University in the United States participated in this study. All participants were recruited from the Department of Communication's research participant pool and received class credit for participating.

Ages ranged from 18 to 28 (Median = 20 y) and 29 subjects were female. Many participants reported prior immersive VR experience (over 90%), curbing any concern that a first-time immersive VR task may impede learning potential (Bricken, 1991). Random assignment was indeed successful as participant age [*t*_(45)_ = −0.47, *p* = 0.64] and gender [$\chi_{\left(1\right)}^{2}$ = 1.18, *p* = 0.28] were evenly distributed across conditions.

### Materials

Consistent with the previous experiment, the immersive virtual experience placed participants underwater on a simulated Mediterranean reef. Depending on condition, participants had one of two different self-avatars: (1) a roughly humanoid-shaped coral, or (2) a scuba diver. The apparatus for this study were consistent with Study 1; both head rotation and head translation were tracked.

### Procedure

**Figure 4:**
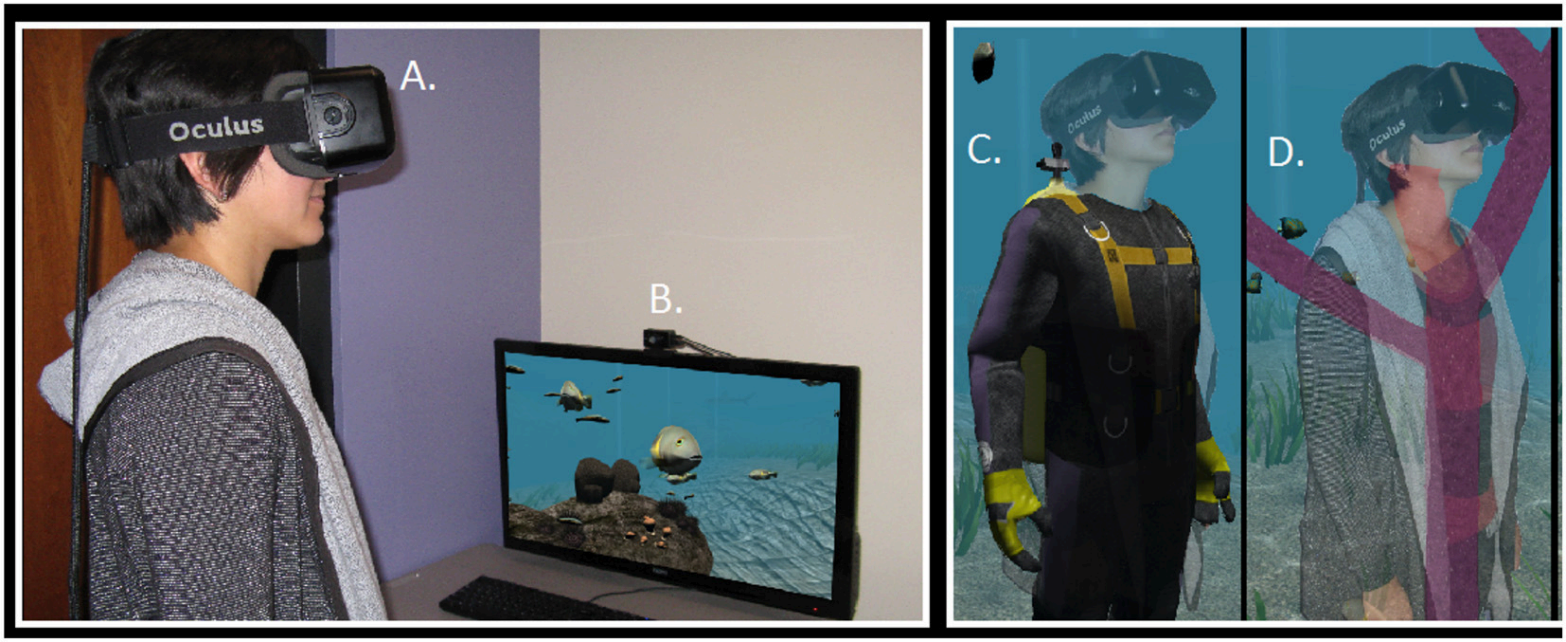
A participant experiencing the virtual environment. **(A)** The participant wears an Oculus Development Kit 2 (DK2) VR display, whose internal accelerometer and gyroscope track 3-degrees of orientation for the user's head. **(B)** The Oculus near infrared (IR) camera tracks IR sensors embedded in the DK2 headset to allow for translational tracking. The IR camera combined with the DK2's accelerometer allows for 6 degrees of freedom tracked on the user's head. **(C)** In the Scuba Diver condition, the user embodies a scuba diver to visit the virtual rocky reef. **(D)** In the Coral Condition, the user embodies a piece of coral that lives on the rocky reef.

Participants were randomly assigned to one of two self-avatar conditions (Coral avatar *n* = 24, Scuba Diver avatar *n* = 23). Besides this change, all experimental conditions were identical (Figure 4 depicts a participant in each experimental condition).

#### Pre-test

Prior to entering the virtual world, participants took a survey consisting of the 14-item Connectedness to Nature Scale (CNS; Mayer and Frantz, 2004), which measures the magnitude of emotional connection to nature and the environment on a scale of (1) strongly disagree, to (7) strongly agree. The seven-point NEP scale from Study 1 (Dunlap et al., 2000), six short answer questions on the causes and effects of ocean acidification, and several demographic measures were also evaluated. Participants were allowed as much time as needed for the pre-test.

#### Immersive VR experience

After the pre-test, participants were introduced to the VR hardware and the remainder of the experiment. The researcher helped participants put on the HMD and headphones, made adjustments to ensure comfort and A/V clarity, and the VR experience began. Participants were standing for the duration of the experiment. The researcher stayed close to the participant and wore headphones to monitor the person's progress through the VR experience.

**Figure 5:**
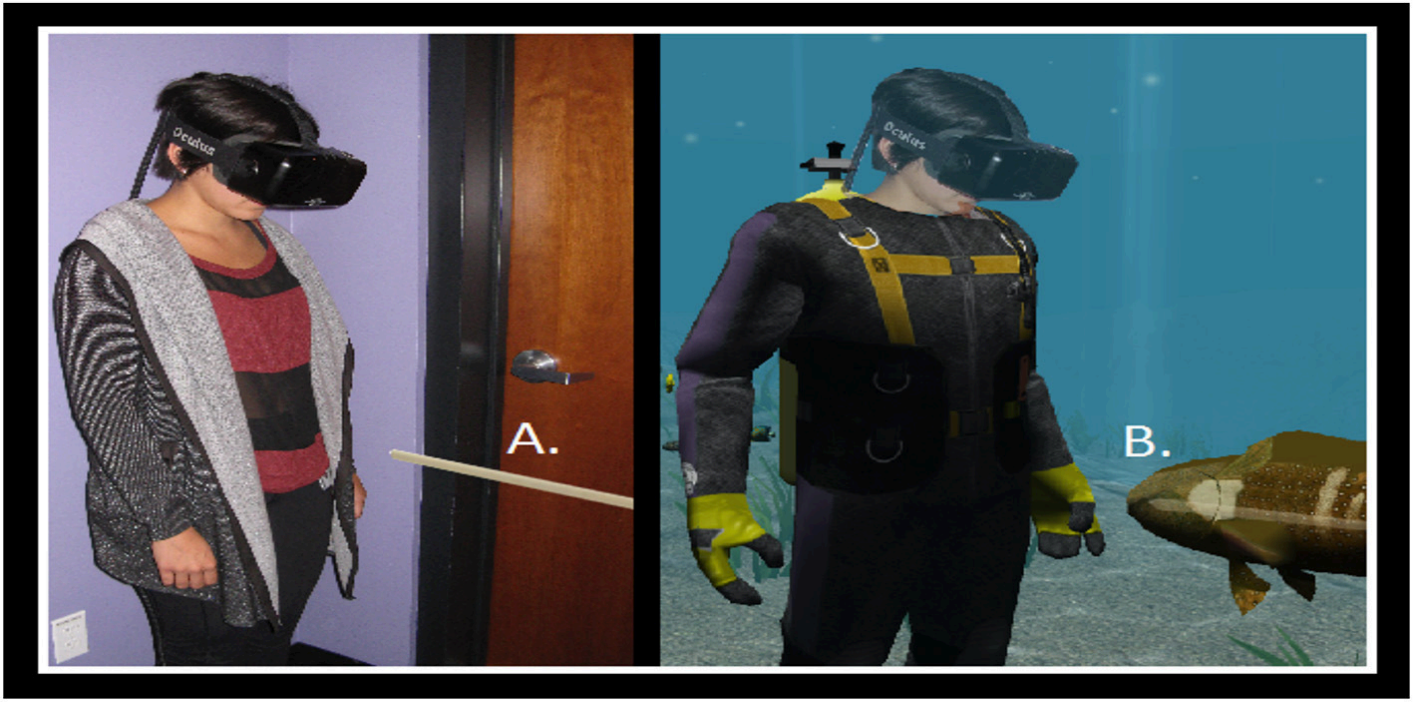
**(A)** The researcher uses a wooden dowel to tap the participant gently in the abdomen. This haptic feedback corresponds with a simultaneous bumping action that occurs in the virtual environment. **(B)** A fish approaches the participant's virtual body and bumps it repeatedly. The fish's bumping action coincides with the researcher's gentle tapping action in the real world.

In the virtual world, participants experienced synchronous environmental feedback designed to make them feel more connected to their self-avatars. Participants saw a fish swim up to their bodies in the virtual world that would begin to repeatedly bump against them. Concurrently, the researcher used a plastic fish on a stick to gently tap the participant on his or her physical body in the same location on their torso as in the virtual world (Figure 5). These taps continued for ~30 s, then the fish swam away. Participants heard an audible “tap” sound as the fish bumped them. This synchronized sensory feedback aims to make participants feel a greater sense of embodiment in their self-avatars (Botvinick and Cohen, 1998; Ehrsson, 2007; Slater et al., 2010). No additional interaction with the researcher occurred. Similar to Study 1, participants did not move or swim in the environment. Participants' position was tracked in the virtual space using the external Rift infrared sensor but they did not physically walk around.

The entire virtual experience took ~6 1/2 min and then participants removed the HMD and headphones. Once subjects confirmed they were not motion sick or otherwise uncomfortable from the virtual experience (none were), they continued to the post-test.

#### Post-test

Participants completed the post-test survey on a computer in the experiment room with no time limit. Post-test measures included a presence questionnaire (adapted from Bailenson and Yee, 2008), the CNS scale, the NEP scale, and six questions from the pre-test about ocean acidification knowledge.

### Measures

#### Ocean acidification knowledge

Responses to questions concerning ocean acidification knowledge were independently scored by two coders, blind to participant condition. Significant differences between graders' scores were rare but resolved after discussion, leading to a high inter-rater reliability (Table 1). In instances where the scores of two raters differed, the average score was used. Scores were summed for each participant, leading to an overall knowledge measure out of twelve possible points.

#### Environmental attitude

Responses to the NEP scale were averaged to create an overall measure of environmental concern (Dunlap et al., 2000).

Items from the CNS scale (Mayer and Frantz, 2004) were highly correlated (Table 1) and averaged. Higher scores reflect more connectedness to nature.

#### Presence

Responses to 9 presence questions were averaged to create an overall presence measure in the virtual environment. Overall, participants reported a moderate level of presence in immersive VR, [(*M* = 5.09, *SD* = 0.73), min = 3.56, max = 6.89].

## Results and discussion

For each measure, we compared scores for the two conditions (Coral vs. Scuba Diver), and when available, compared pre- and post-test scores. We also examined correlations among dependent variables to test the relationship between knowledge, attitudes, and presence.

### Ocean acidification knowledge

A repeated measures ANOVA calculated the change in ocean acidification knowledge across condition and from the pre-test to post-test. The difference in ocean acidification knowledge from the pre-test [*M* = 2.51, *SE* = 0.26; 95% CI (1.99, 3.03)] to post-test [*M* = 8.25, *SE* = 0.31; 95% CI (7.61, 8.88)] was statistically significant, [*F*_(1, 46)_ = 290.23, *p* < 0.001, $\eta_{\text{p}}^{2}$ = 0.86], but there was no difference in knowledge gain across Coral and Scuba Diver conditions [*t*_(45)_ = 0.06 *p* = 0.95, *d* = 0.02].

### Environmental attitude

For the NEP scale, the difference between pre-test [*M* = 4.77, *SE* = 0.10; 95% CI (4.56, 4.98)] and post-test [*M* = 5.16; *SE* = 0.10; 95% CI (4.95, 5.37)] scores was significant [*F*_(1, 46)_ = 78.12, *p* < 0.001, $\eta_{\text{p}}^{2}$ = 0.63], but the change was not significantly different across conditions [*t*_(45)_ = 0.33, *p* = 0.75, *d* = 0.10]. For the CNS scale, there was a positive attitude change from pre-test [*M* = 4.45, *SE* = 0.11; 95% CI (4.23, 4.66)] to post-test [*M* = 4.71, *SE* = 0.12; 95% CI (4.46, 4.95)], [*F*_(1, 46)_ = 18.66, *p* < 0.001, $\eta_{\text{p}}^{2}$ = 0.29], but again, no significant difference in attitude change on the CNS scale across condition [*t*_(45)_ = 0.23, *p* = .82, *d* = 0.07].

### Presence

There was no significant difference in the reported feeling of presence between the Coral (*M* = 5.00, *SD* = 0.70) or Scuba Diver (*M* = 5.18, *SD* = 0.76) experimental conditions [*t*_(45)_ = −0.84, *p* = 0.41, *d* = 0.24], nor did we find differences in responses to the individual presence items across conditions.

#### Correlations between measures

There were several significant, positive relationships between post-test scores (see Table 3). Presence was positively associated with post-test levels of ocean acidification knowledge (ρ = 0.34, *p* = 0.019), ratings on the CNS (ρ = 0.47, *p* = 0.001) and NEP scales (ρ = 0.46, *p* = 0.001). Therefore, the more that people reported being attuned to the virtual environment in the post-test, the more that they learned in immersive VR, felt connected to nature, and reported environmental concern. Correlations between measures that are calculated based on the change between pre- and post-test scores can be found in Appendix B; as expected, they show largely the same pattern as post-test correlations alone.

**Table 3:** Correlation Matrix: Study 2 (Two-tailed, *N* = 47).

	**OAK**	**CNS**	**NEP**	**Presence**
OAK	—	0.05	0.31^*^	0.34^*^
CNS	0.05	—	0.33^*^	0.47^***^
NEP	0.31^*^	0.33^*^	—	0.46^***^
Presence	0.34^*^	0.47^***^	0.46^***^	—

*Correlations were computed using Spearman's rho*.

* *p < 0.05*,

*** *p < 0.001. OAK, Ocean Acidification Knowledge; CNS, Connectedness to Nature Scale; NEP, New Ecological Paradigm scale. Correlations were computed using OAK, CNS, NEP, and presence scores in the post-test*.

Replicating the results from Study 1, participants displayed positive learning about marine science in immersive VR in a controlled laboratory setting. We explored the possibility that embodying a coral vs. a human scuba diver would lead to more knowledge gain, positive attitude toward the environment, and greater levels of reported presence, though consistent levels of these measures were observed across experimental conditions. Crucially, the data suggest that learning and an increase in environmental concern can occur regardless of the avatar that a person embodies. These findings substantiate and extend the results from Study 1 by exploring the learning effects in an environment with considerably less empirical noise. It could be possible that the significant effect on environmental attitude appeared with a more robust sample size relative to Study 1.

Despite the demonstrable learning effects from the pre-test to the post-test, it is important to consider potential explanations for the null effect across self-avatar conditions. One possibility is that the virtual world provided too rich of an experience and the manipulated self-avatar was not the most salient stimuli in the environment. It is also possible participants ignored the virtual environment and listened to the narration, or vice versa, leading to a dissociation of attitude change and learning gains. However, we observed that participants' reported levels of presence, or the feeling that the virtual world was unmediated, was positively associated with post-test learning scores. Since we observed this finding in a *post-hoc* test, we went back into the field to understand other how other aspects of a virtual experience can predict knowledge gain. In an exploratory field study with adults, we tracked how physical arm or head movements may relate to an interest in learning about ocean acidification since prior work suggests that interactivity in media can produce positive associations with a target and facilitate deep cognitive processing (Tremayne and Dunwoody, 2001; Sundar, 2012).

### Study 3

This field study was conducted at the 2016 Tribeca Film Festival, a 5 days event that hosts films, documentaries, and games to be experienced by attendees. People at the festival visit various exhibits on the floor, and in our case, the “Virtual Arcade.” The general virtual experience, similar to the one that participants encountered at the Tribeca Film Festival, is publicly available (https://store.steampowered.com/app/409020/The_Stanford_Ocean_Acidification_Experience/).

### Method

#### Participants

In the 5 days of the Tribeca Film Festival, 488 people participated the immersive VR experience either wholly or partially, and several participant exclusion criteria were used. First, we discarded data collected from the 1st day due to general technical failures. Data from participants were also excluded if the individual did not comply with the study's task (e.g., if he or she swam so far outside the simulated reef that they missed the interactive simulation). Finally, we excluded participants who failed to respond to our attitude prompts in the post-underwater experience. This reduced our sample size to 167 participants, though this number remains large enough to possibly detect an effect of physical movements on interest in learning about ocean acidification.

Due to time constraints set by the festival—to have an exhibit, virtual experiences were required to be <10 min long—we did not formally measure demographics of participants. In general, however, there was greater age and demographic variance in this study than a typical sample of university undergraduates.

#### Materials

For this study, we used the HTC Vive with a resolution of 2160 × 1200 and an update rate of 90 Hz (e.g., 90 frames per second, per eye), tracked at a 22 ms latency rate. Two tracking base stations provided a six degrees-of-freedom control each for the head (through sensors embedded inside the Vive) and the two hands (through hand controllers; 120 Hz update rate, 8.333 ms latency). Participants also received audio feedback provided by stereo headphones. We used Worldviz's Vizard for developing the VR experience and used the 3D models developed by Worldviz around Italy's Ischia Reef. The 3D environment modeled the Mediterranean rocky reefs located off the coast of Ischia, Italy, which were simulated in Studies 1 and 2. The virtual environment in Study 3 was similar, but not identical to the virtual environment in Study 2. Slight modifications were made to the virtual environment due to affordances granted by more advanced hardware and computing power. The modifications included: increasing the fidelity of the graphics and the size of the virtual space that could be explored (e.g., participants could move and interact more within the environment).

The immersive VR stations were 10' × 10' booths with four curtained walls. It should be noted, however, that the size of the virtual space exceeded physical space limitations. We encouraged participants to stand in one place, swim by moving their arms in a breaststroke motion, and rotate to change their direction.

#### Procedure

The experiment, including consent procedure, was a self-contained 8 min experience. The embedded informed consent procedure was approved by our university's Institutional Review Board. Pre-recorded narratives for this field trip, recorded by an expert voice-actor, were played through noise-canceling stereo headphones and guided the participants throughout the experience. To display text and visual information, we used virtual information boards. Participants responded to the experimental questions by placing their hands inside virtual choice-boxes and clicking a button on the HTC Vive controllers to select answers to written prompts that appeared within the virtual environment.

**Figure 6:**
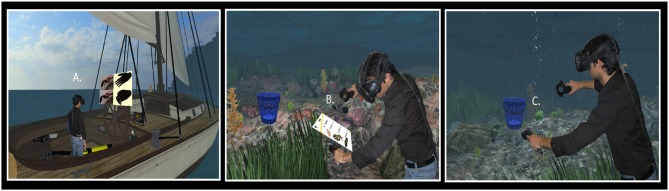
**(A)** The participant begins the experience by listening to an introductory narration and watching short tutorials that describe how he will interact with objects in the underwater environment. **(B)** The participant engages in a “species count.” He refers to the dive slate attached to his virtual left wrist to see which species are on the list, how many he has found already, and how much time is left. The participant is given 2 min to find species in the first healthy underwater zone. **(C)** The participant is transported to an unhealthy zone with higher levels of acidity. He is given another 2 min to conduct a species count in this area.

The VR experience began on a virtual boat floating above an Ischian Reef (see Figure 6). Participants first viewed and responded to the informed consent. A series of narratives and infographics then trained each participant on their task, which was to swim underwater by moving their arms in a breaststroke motion and find marine life to measure the biodiversity of the reef. The immersive VR scene brought participants to the first underwater zone, modeled around a healthy rocky reef. Participants were asked to locate three different underwater animal species (snails, octopi, and eel) as part of a “species count,” an activity modeled after biodiversity assessments that marine biologists conduct in the field. For the next 2 min, participants used their arms to swim around the virtual underwater reef looking for the species of animals and collecting as many as they could find by picking them up with their hand controllers and placing them into a bucket located at the reef's center. A dive slate—modeled after the slates used by marine biologists—attached to each participant's virtual left wrist, displayed the remaining time, and how many of each species had been collected.

After 2 min, participants were moved to an acidified zone and asked to perform the same species collection task, however, the acidified zone only contained grass and degraded snails. This subtly communicated the message about the ill effects of ocean acidification, implying that the healthy species could no longer survive in an acidified environment. After counting species in the acidified zones, participants returned to the boat and compared the number and the variety of species in the two zones on an infographic. The narration then told participants about the general consequences of ocean acidification around the world. Participants then responded to an attitude and inquisitiveness question related to ocean acidification, which were presented inside the VR world, and then the experience ended with credits and acknowledgments.

#### Measures

Given that the measures for this study were administered during the immersive VR experience and the focus of the venue was not on experimental studies, we avoided standardized questionnaires such as those used in our previous experiments. Instead, we created two simpler dependent variables to measure each participant's attitude toward the immediacy of ocean acidification and inquisitiveness about the issue at large.

##### Movement

We tracked the head and hand movements of participants during the species count, which included working with the virtual slate, to discover if the amount of movement predicted attitude or inquisitiveness. Descriptive statistics for head and hand movements are located in Appendix C.

##### Attitude

After the immersive VR experience ended, participants responded to the question, “How far away do the consequences of ocean acidification feel to you?” with five answer choices: (1) extremely far, (2) far, (3) reasonable time remains, (4) close, and (5) extremely close. The median response was higher than the midpoint (4), with a slightly smaller mean level of concern for the consequences of ocean acidification [(*M* = 3.53, *SD* = 0.97), min = 1.0, max = 5.0].

##### Inquisitiveness

After the attitudinal question, participants answered yes or no to the question, “Would you like us to send you more information about ocean acidification?” Approximately one-third of participants asked for more information (53/167; 31.7%).

##### Species count and search activity

We counted the number of species that each participant grabbed during the underwater experience [(*M* = 10.22; *SD* = 14.17), min = 0, max = 116.0]. This measure differed, however, from the number of species that participants put into their collection buckets [(*M* = 3.90; *SD* = 3.57), min = 0, max = 21]. These items were highly correlated (ρ = 0.74, *p* < 0.001) and we therefore combined these scores into a standardized index called *search activity* to evaluate participant overall interactivity level during the species count.

### Results and discussion

We assessed attitude toward ocean acidification and inquisitiveness based on head and hand movements, which are evaluated as separate predictors. We used movement to predict attitude and inquisitiveness because we were interested in exploring physical actions that may be indicative of environmental concern and if people who move more in the virtual space are also likely to express greater interest in environmental issues (Clark, 1997; Gallagher, 2005). For each regression model, we include physical movement and physical rotation of the head and hands as separate predictors because changes to these body parts are often concurrent. That is, movement of the head and hands is also accompanied by rotation of the head and hands (ρ = 0.64, *p* < 0.001; Table 4).

**Table 4:** Correlation Matrix: Study 3 (Two-tailed, *N* = 167).

	**Inquisitiveness**	**Attitude**	**Total movement**	**Total rotation**	**Search activity**
Inquisitiveness	—	0.19^*^	0.10	−0.05	0.06
Attitude	0.19^*^	—	−0.05	−0.10	−0.10
Total movement	0.10	−0.05	—	0.64^***^	0.34^***^
Total rotation	−0.05	−0.10	0.64^***^	—	0.47^***^
Search Activity	0.06	−0.10	0.34^***^	0.47^***^	—

*Correlations were computed using Spearman's rho*.

* *p < 0.05*,

*** *p < 0.001. Inquisitiveness was a dichotomous variable (1 = would like more information, 0 = would not like more information). Search activity is a standardized index of the grabbed and collected biodiversity during the species count*.

We used a layered analytic approach to isolate if movement or rotation predict attitude and inquisitiveness. First, we combined the total movement of the head and two hands into a single predictor, *total physical movement*, and the total rotation of the head and two hands into a single predictor, *total physical rotation*. If statistically significant, these global variables were then isolated by body part (head, two separate hands) to discover how movement and rotation of specific body features predict attitude and inquisitiveness.

#### Attitude

Total physical movement and total physical rotation did not predict participants' attitude toward the immediacy of ocean acidification [*p*'s > 0.40]. Therefore, we did not inspect this effect further.

#### Inquisitiveness

A logistic regression model predicting participant inquisitiveness (1 = would like more information, 0 = would not like more information) with total physical movement and total physical rotation as standardized predictors showed a significant effect of total physical movement [(β = 0.45, *SE* = 0.23); Wald = 3.90, *p* = 0.048, Exp(β) = 1.57; 95% CI for Exp(β) [1.00, 2.47], but no effect of total physical rotation [*p* = 0.12, Exp(β) < 1; 95% CI for Exp(β) [0.44, 1.10], Nagelkerke *R*^2^ = 0.03].

Consistent with our layered analytic approach, we then ran three separate logistic regression models for each body part's movement (e.g., head, left hand, right hand) and concurrent rotation as independent predictors. The data revealed that the total physical movement of the participant's right hand, proxied by the movement of the virtual right-hand model, significantly predicted inquisitiveness [(β = 0.54, *SE* = 0.25); Wald = 4.74, *p* = 0.029, Exp(β) = 1.71; 95% CI for Exp(β) [1.06, 2.77]. Physical total rotation of the right hand was marginally associated with inquisitiveness, [*p* = 0.069, Exp(β) = 0.64; 95% CI for Exp(β) [0.39, 1.04], Nagelkerke *R*^2^ = 0.04]. Movement and rotation of the left hand were not significant predictors of inquisitiveness [*p*'s > 0.19, Exp(β) < 1.32; Nagelkerke *R*^2^ = 0.02], and neither were movement or rotation of the head [*p*'s > 0.18, Exp(β) < 1.27; Nagelkerke *R*^2^ = 0.02].

#### *Post-hoc* correlations

Based on the prior evidence suggesting that right-hand movements can predict inquisitiveness, we analyzed the data for other *post-hoc* relationships. The point-biserial correlation between inquisitiveness and attitude toward the closeness of ocean acidification revealed a positive relationship (ρ = 0.19, *p* = 0.014). The exact direction of this effect is unclear, however, because it is plausible that the more people believe climate change is an immediate threat, the more they may be interested in learning about the issue. On the other hand, if people report being inquisitive about climate change, they may also feel like the issue is impending. Further research is required to understand this effect more clearly. Finally, we found that participants' total body movement (ρ = 0.34, *p* < 0.001) and rotation (ρ = 0.47, *p* < 0.001) were significantly correlated with total search activity (e.g., total species grabbed and collected in the species count). All correlations for this study's key variables are located in Table 4.

The data suggest that participant right hand movements can predict if people want to learn more about climate change. How can right hand movements predict inquisitiveness? Prior work suggests that people often process information differently based on the side of their physical body that is completing an action (Casasanto, 2011; Bailey et al., 2016). This idea is called processing fluency (Reber et al., 2004), with evidence suggesting that making one hand inaccessible (e.g., wearing a bulky ski glove on one hand) changes how people perceive the space (e.g., the side that is accessible is viewed more positively than the side that is inaccessible; Casasanto and Chrysikou, 2011). It is plausible that people with more right-handed movements also were inquisitive because they enjoyed the virtual experience and formed a positive association with it. This contention is worth exploring more directly in future work.

There are two primary limitations of this study worth noting. First, we did not gauge handedness of the participants. Second, people at the Tribeca Film Festival paid $40 to get into the VR arcade and stood in line for our immersive VR booth throughout the week-long festival. This information suggests that subjects who participated in our study may therefore have been extrinsically motivated to acquire knowledge about novel experiences in general. Given that we have some evidence that movement can predict interest in learning about climate change, albeit data from an uncontrolled field study, we tested the robustness of these effects in a more sterile laboratory environment with university undergraduates.

### Study 4

We did not have granular data from Study 3 on the types of movements that predicted inquisitiveness with the right hand. Therefore, to explore how other movements can predict knowledge gain and attitude change about ocean acidification, we tested movements that are reasonably germane to underwater swimming that may affect learning. Specifically, we evaluated if knowledge gain and attitude change are affected by two locomotion techniques: (1) swimming by using physical arm movements as gestures, and (2) swimming by using a joystick (e.g., the Wiimote, based on the Nintendo Wii gaming console).

### Method

#### Participants

Forty-four students from a large West Coast University in the United States participated in the experiment for course credit. One participant was excluded due to hardware failures, leaving 24 females and 19 males in the final sample.

**Figure 7:**
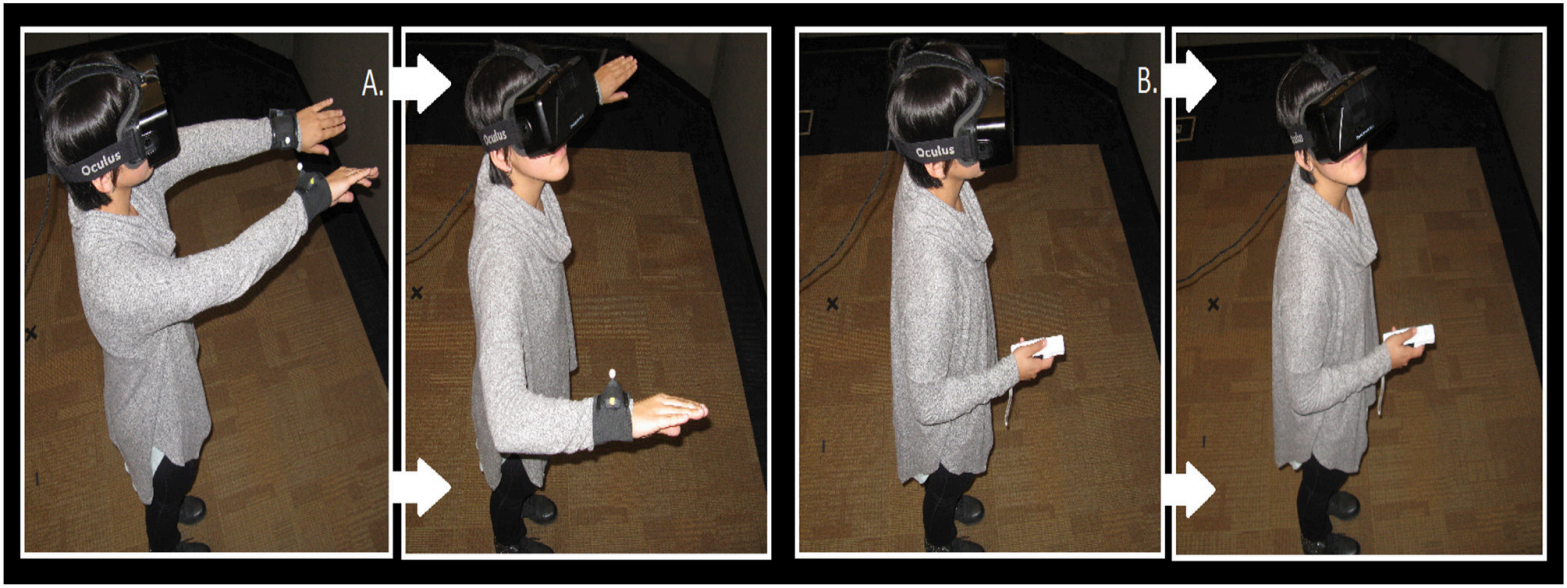
**(A)** In the Gestures condition, the participant is outfitted with Worldviz PPT-E infrared sensors attached to her wrists, to allow her to swim through the virtual ocean. The participant moves her arms in a breast-stroke-like motion to swim forward through the environment and turns her head to change direction. **(B)** In the Joystick condition, the participant uses a Wiimote controller to swim through the environment. She presses the arrow keys on the Wiimote controller to control the direction in which she swims and can turn her head to change directions.

A total of 21 participants swam using the remote control, henceforth called the Joystick condition, and 22 participants swam using their physical gestures, henceforth called the Gestures condition. Figure 7 shows a participant in each condition. Random assignment was indeed successful for this study as participant age [*t*_(41)_ = −0.78, *p* = 0.44] and gender [$\chi_{\left(1\right)}^{2}$ = 0.29, *p* = 0.86] were evenly distributed across conditions.

#### Materials

The immersive VR HMD for this study was consistent with Study 1 and 2, and the virtual reef was identical to Study 3. An optical tracking system from Worldviz (PPT-E; Figure 7), with 180 Hz update rate and 20 ms latency, provided six degrees-of-freedom positional tracking each for the head and the hands. A 24-channel Ambisonic Auralizer Sound System provided spatialized audio feedback to the participants. We used this audio feedback method since it was the default setting for the laboratory. Consistent with Study 3, we used Worldviz's Vizard software to create the VR experience and Worldviz's 3D models of the Mediterranean rocky reef.

The physical space was a 12' × 17' laboratory room. The virtual space was larger than the physical space, however. We encouraged participants to stand in one place and rotate their body to change their direction. Participants in the Joystick condition navigated the underwater world with a hand-held device that had both discrete buttons and continuous pad to navigate the underwater world. Participants pressed the four edges of the continuous pad on the Wiimote to navigate in cardinal directions. All input choices were communicated through Bluetooth.

Participant in the Gestures condition performed a breaststroke swimming motion to navigate the underwater world. Each breaststroke motion moved the participant forward in the direction they were facing and movements were discrete accelerations. Decelerations were not representative of actual water resistance, but instead, were empirically derived and constant to minimize nausea from the underwater navigation.

#### Procedure

Participants first read and signed a consent form. Then, they completed a pre-test on a laptop, which solicited demographic information, current knowledge about ocean acidification, and the participant's estimate of the current temperature of the room.

The experimenter explained the immersive VR hardware for viewing and interacting with the virtual world. Depending on the participant's experimental condition for moving underwater, they were provided with Wiimote controls or given instructions for swimming with their hands. Participants then wore the HMD and began their VR experience. A pre-recorded narrative guided subjects throughout the experience at an adjusted level of ambient audio clarity. To ensure familiarity with the underwater world, participants had a 1 min training session to practice locomotion techniques and collect flora and fauna in a virtual underwater zone. Subjects also answered a question that asked about the current temperature since the last time they responded, an exploratory measure that assessed if exposure to climate change effects can modify perceptions about one's environment.

**Figure 8:**
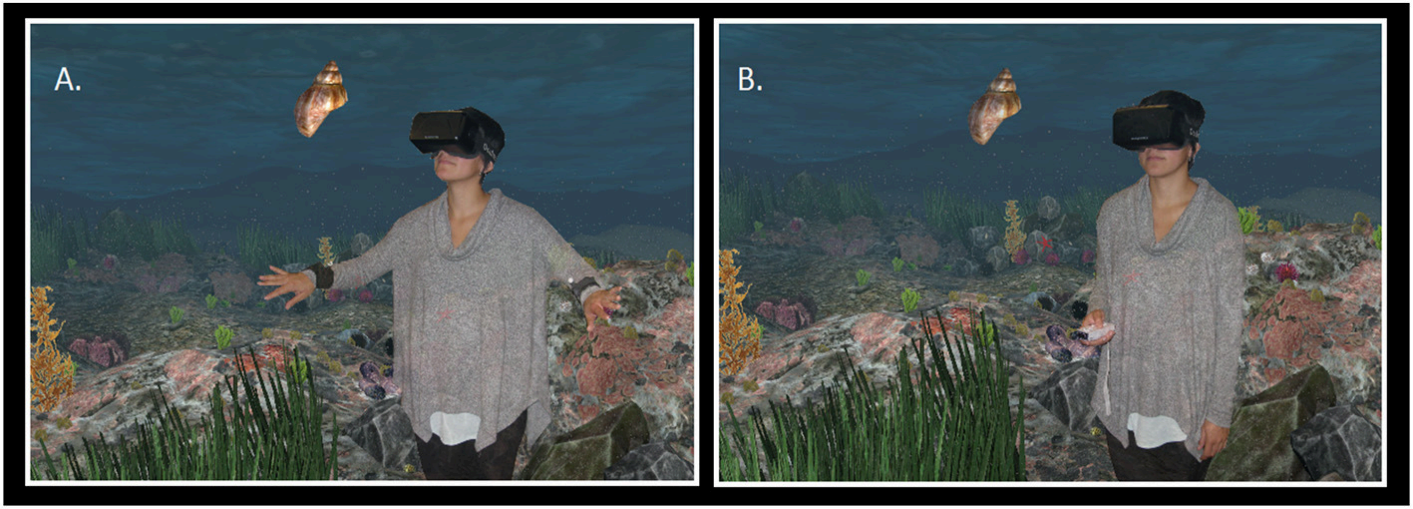
**(A)** The participant is shown an example of the type of species she will be looking for before she begins her species count on the virtual reef, using her arms to swim. **(B)** The participant is shown an example of the type of species she will be looking for before she begins her species count on the virtual reef, using the Wiimote controller to move throughout the environment.

The experimenter then placed the participant in the healthy underwater zone. A narrative explained that the virtual world was modeled around a Mediterranean rocky reef, with a colorful and diverse ecosystem of underwater plants and animals. Participants were also shown sea snails, the species they would be searching for and counting in this zone. Figure 8 shows how the 3D model of the snail appeared to participants in each of the conditions.

At each stage of the underwater experience, the negative consequences of ocean acidification and climate change became more apparent and exacerbated. First, subjects searched for and counted as many snails as they could find for 2 min, using their condition-specific locomotion technique, and answered a question about the current temperature since the last time they reported. This first zone is henceforth referred to as the *High pH*, or low acidification zone. The experimenter then placed participants in a second, climate change affected zone, henceforth referred to as the *Medium pH*, or moderate acidification zone. The narrative informed subjects that volcanic vents were releasing carbon dioxide in the water and making the water slightly more acidic, a process called ocean acidification. Comparisons were drawn between the effect of the volcanic vents and the effect of human-released carbon dioxide through the burning of fossil fuels. Descriptions about ocean acidification and its undesired changes in ocean plants and animals were also provided. Participants went on another search for snails and answered a question about the current temperature since the last time they responded. Last, participants moved to the most acidic underwater zone, henceforth referred to as the *Low pH*, or high acidification zone. The narrative described that the zone composition reflected scientific predictions of what the earth's oceans will look like in 100 years if fossil fuels continue to be burned at the current rate. In this last zone, participants answered a question about the current temperature since their last response, but did not search for snails as there were none to find.

After the *Low pH* zone, the researcher helped participants remove the HMD, trackers (in the Gestures condition), and subjects completed a post-experience questionnaire on a laptop. Participants were debriefed and then dismissed after completion of these items.

#### Measures

Consistent with our prior studies, we asked questions about ocean acidification and administered the CNS, NEP, and presence scales. We also evaluated the total distance traveled underwater as a gauge of interactivity in the virtual environment. Finally, other exploratory measures such as the perceived room temperature and ratings of simulator sickness (Kennedy et al., 1993) were investigated (for these ancillary measures, see Appendix D).

##### Ocean acidification knowledge

Participants answered three questions about ocean acidification and their responses were judged by three blind raters. The questions asked, “What is ocean acidification?,” “What are the causes of ocean acidification?,” and “What are the predicted effects of ocean acidification?” Following the procedure established in Study 1, questions received grades of 0, 1, or 2 based on their response accuracy. Questions were randomized in the judgment process to control for order effects. Ideal answers are located in Appendix E.

##### Attitude

Participants responded to two primary attitude questions. The first question asked how far away the consequences of ocean acidification felt, on a scale from (1) extremely far, to (5) extremely close [(*M* = 3.33, *SD* = 1.02), min = 1.0, max = 5.0]. The second question consisted of six items where on five-point scales, participants responded to the consequences of ocean acidification, ranging from (a) unimportant to important (b), of no concern to of concern (c), irrelevant to relevant (d), means nothing to means a lot (e), doesn't matter to matters a lot (f), uninvolving to involving (Cronbach's α = 0.94). Overall, participants reported an above-average level of concern for ocean acidification, [(*M* = 4.05, *SD* = 0.74), min = 1.67, max = 5.0]. These two measures were evaluated separately and also averaged to create an overall attitude score toward ocean acidification [(*M* = 3.69, *SD* = 0.80), min = 1.83, max = 5.0; Cronbach's α = 0.78].

##### CNS and NEP scales

Participants responded to the CNS, [(*M* = 3.36, *SD* = 0.53), min = 2.36, max = 4.79], and NEP scales, [(*M* = 3.72, *SD* = 0.58), min = 2.33, max = 4.80]. For this study, the CNS and NEP scales were measured from (1) strongly disagree to (5) strongly agree.

##### Presence

Self-reported presence was the averaged response to five, five-point Likert scale questions, [(*M* = 3.16; *SD* = 0.92), min = 1.60, max = 4.80] in the post-survey that measured the degree to which participants felt surrounded by the virtual environment.

##### Total number of snails found

We tracked the number of snails found in the *High pH* and *Medium pH* zones as an indicator of activity level. On average, participants found over 23 snails across the two zones combined, [(*M* = 23.74; *SD* = 3.87), min = 13.0, max = 29.0].

##### Distance traveled underwater

We also tracked the participants' viewpoint through the virtual world, which let us calculate the total distance traveled underwater across three zones, [(*M* = 128.12 m; *SD* = 37.17 m), min = 45.49 m, max = 193.26 m].

### Results and discussion

We report no effect of locomotion technique on the separate or overall attitude measures, the CNS scale, the NEP scale, reported temperature change, presence, and simulator sickness, [*t*'s <1.58, *p*'s > 0.12]. It is possible that these null effects emerged because the locomotion techniques were not distinct enough to establish differences on perceptions-based dependent measures. That is, participants could travel in generally the same path across conditions but their approaches were dissimilar. Such contrasts may not be strong enough to affect participants, or perhaps the data reveal that psychological effects overwhelm physical effects in VR. The null main effects are not discussed further due to space limitations.

### Ocean acidification knowledge

A repeated measures ANOVA revealed a significant improvement of ocean acidification knowledge scores after comparing pre- [*M* = 2.70, *SE* = 0.24; 95% CI (2.22, 3.18)] and post-test scores [*M* = 3.63, *SE* = 0.14; 95% CI (3.35, 3.91)], [*F*_(1, 42)_ = 22.95, *p* < 0.001, $\eta_{\text{p}}^{2}$ = 0.35]. There was no main effect of locomotion technique on the change in knowledge scores, however [*p* = 0.73, *d* = 0.11].

### Number of snails found

As expected, a repeated measures ANOVA revealed that participants found more snails in the *High pH* zone [*M* = 15.35, *SE* = 0.41; 95% CI (14.52, 16.18)] than the *Medium pH* zone [*M* = 8.40, *SE* = 0.24, 95% CI (7.92, 8.87)], [*F*_(1, 42)_ = 474.82, *p* < 0.001, $\eta_{\text{p}}^{2}$ = 0.92]. The change in the number of snails found did not differ across locomotion methods, however [*p* = 0.11, *d* = 0.50].

### Distance traveled underwater

We used hierarchical linear mixed models with two fixed effects (Zone, three levels; Condition, two levels) and a random effect for participant (to control for multiple observations by the same individual) to determine distance traveled by participants underwater. We found a main effect of Zone, [*F*_(2, 84)_ = 10.38, *p* < 0.001, η^2^ = 0.18]. *Bonferroni*-corrected adjustments revealed that participants traveled more in the *Low pH* zone (*M* = 47.70 m, *SE* = 2.20 m) than the *Medium pH* zone [(*M* = 41.18 m, *SE* = 2.20 m); *p* = 0.004] and the *High pH* zone [(*M* = 39.24 m, *SE* = 2.20 m); *p* < 0.001]. There was no difference in distance traveled across the *High pH* and *Medium pH* zones, however (*p* > 0.80). There was also no significant main effect of locomotion technique on distance traveled [*F* < 1, *p* > 0.50] and no interaction between locomotion technique and zones for distance traveled [*F*_(2, 82)_ = 0.62, *p* = 0.54, η^2^ = 0.17].

### *Post-hoc* correlations

Consistent with the evidence in Study 2, Table 5 suggests that participants' self-reported presence was significantly correlated with CNS scores (ρ = 0.32, *p* = 0.035) and NEP scores (ρ = 0.41, *p* = 0.007). Further, reported levels of presence were positively correlated with the average attitude measure (ρ = 0.44, *p* = 0.004). These data suggest that the more the participants felt they were a part of the underwater experience, the stronger they felt concerned about the effects of climate change and ocean acidification.

**Table 5:** Correlation Matrix: Study 4 (Two-tailed, *N* = 43).

	**OAK**	**CNS**	**NEP**	**Presence**	**Items collected**	**Simulator sickness**	**Distance**
OAK	—	−0.07	−0.13	−0.04	0.35^*^	0.02	0.40^**^
CNS	−0.07	—	0.50^**^	0.32^*^	0.13	0.21	0.01
NEP	−0.13	0.50^**^	—	0.41^**^	0.001	0.32^*^	−0.07
Presence	−0.04	0.32^*^	0.41^**^	—	0.004	0.31^*^	−0.13
Items collected	0.35^*^	0.13	0.001	0.004	—	0.05	0.14
Simulator sickness	0.02	0.21	0.32^*^	0.31^*^	0.05	—	−0.13
Distance	0.40^**^	0.01	−0.07	−0.13	0.14	−0.13	—

*OAK, Ocean Acidification Knowledge (e.g., the learning change from pre- to post-test ocean acidification scores), CNS, Connectedness to Nature Scale; NEP, New Ecological Paradigm scale. Correlations were computed using Spearman's rho*.

* *p < 0.05*,

** *p < 0.01. Items collected considers the number of snails collected across all zones. Distance considers the total distance traveled in the underwater world*.

Crucially, participants' knowledge improvement about ocean acidification (e.g., the change from pre- to post-test ocean acidification knowledge scores) was positively correlated with the total number of snails found across zones (ρ = 0.35, *p* = 0.022) and total distance traveled underwater (ρ = 0.40, *p* = 0.008), suggesting that visual exploration and overall activity may lead to greater change in ocean acidification knowledge. This potential mechanism is discussed below.

## General discussion

We present four studies exploring the effect of an immersive VR experience on learning about marine science, specifically the consequences of climate change. All four investigations reached the same general conclusion: after an immersive VR experience, people report positive knowledge gain or an interest in learning about the causes and effects of ocean acidification. In some cases, we observed pro-environmental attitude change and *post-hoc* analyses uncovered a potential mechanism for the increased learning effect in Study 4. Participants who explored, traveled, and found more marine objects also demonstrated greater change in learning from pre- to post-test scores about climate change. Our work shows that learning gains or an interest in learning can occur across different participants (high school students, college students, adults), measures (learning gain data, tracking data), and content (versions of the same ocean acidification topic were created).

There are several distinct contributions of this paper that go beyond the reported learning effects. First, Study 1 serves as an initial proof of concept that immersive VR can deliver complex scientific information to young adults in classrooms. Increased learning likely occurred because the abstract and distant effects of ocean acidification were brought psychologically closer to participants (Trope and Liberman, 2010). We created immersive VR experiences that brought oceanic and marine life transformations to students in a manner of minutes, plausibly changing their views on the immediacy of ocean acidification and climate change from conceptually abstract to concrete.

Second, we address a need in the environmental education literature by bringing innovative content and experiences to climate change education (McCright et al., 2013; Wu and Lee, 2015). Our studies show that people can learn about complex social and environmental issues when information is brought to them in an immersive format. Learning also does not appear to be a momentary artifact, as our longitudinal design (Study 1) suggests that people can retain information about ocean acidification weeks after being in immersive VR. Since our paper presents data on participants not limited to college student samples, we are optimistic that these learning patterns are robust and not constrained by age, one's familiarity with new media, or the compensation that he or she received for research participation.

Our studies also describe the parts of an immersive VR experience that may be difficult to manipulate in a learning setting. Perhaps, our avatar and locomotion manipulations failed to modify the psychological experience of our participants because the focus on exploring and learning about complex information was crucial to the procedure. If the learning content is more familiar to subjects at the study's onset, this may allow participants to focus on their character in a virtual scene and demonstrate learning.

## The relationship between immersive VR and climate change education

What do our studies reveal about the relationship between media and learning? The Interactive Information Processing Model by Tremayne and Dunwoody (2001) suggests when people use media interactively, they engage in more elaborative cognitive processing than people who use media passively. For example, people who use interactive websites that allow clicking on links engage in deeper cognitive processing and appraise their mediated experience more positively than people who use sites that only allow scrolling (Sicilia et al., 2005; see also Xu and Sundar, 2016). Therefore, participants who explored more of the virtual space formed deeper cognitive associations with the science content and could learn, recall, and retain the causes and effects of ocean acidification better than those who did not explore the underwater world as much. We suspect that the strong learning effects in immersive VR reflect the medium's unique ability to fully engage a subject in an experience and surround him or her with the feeling of non-mediation (Slater and Wilbur, 1997), but also believe that it is important to explore how immersive VR compares to other media in its potential to facilitate learning about science.

We observed that the change in knowledge about ocean acidification was linked to increased engagement with and exploration of the virtual space (Study 4). This *post-hoc* mechanism is further supported by Sundar (2012), who suggests that increased interactivity demands more conscious and deliberate processing of mediated content for comprehension. The implications of interactivity have been demonstrated largely with websites and online media, though the conceptual underpinnings of this phenomenon are transferrable across media experiences (Sundar et al., 2013). Therefore, we pre-suppose that moving within the space that looked and behaved like a real underwater reef made it easier for people to elaborate and understand the consequences of climate change, and the more that people explored in VR the more they benefited from the virtual experience. This pattern may serve as one recommendation for teachers and immersive VR developers who want to build virtual effective content for classrooms.

## Challenges, limitations, and future directions

Deploying VR hardware in a classroom presented one unforeseen challenge: wearing a HMD hides the physical world from a user, but it does not hide the user from the physical world. Interacting with a world that is invisible to those outside the HMD makes a user look strange to an observer, a fact that became clear in our classroom work (Study 1). Teachers had to remind students of school policies on social media use, as students not engaged in the VR experience were taking pictures of their peers wearing HMDs and attempting to share those pictures online.

There are general limitations across studies that are also worth highlighting and resolving in future work. First, it is unclear how long the learning effects may last in most of our studies. Information retention is an issue for most examinations that evaluate technology for education (see Mikropoulos and Natsis, 2011). Although we encourage future work to explore the potential of immersive VR to increase retention rates over time, our results offer a hopeful outlook, given that weeks after an underwater experience, high school students retained knowledge about ocean acidification.

In addition to exploring information retention, immersive VR should be compared to other media to evaluate the boundary conditions for learning about marine science. Some research suggests that learning about science through a HMD produces similar learning levels as desktop VR (Moreno and Mayer, 2002), but it is important to position immersive VR relative to other innovative teaching platforms and approaches. For example, it is unclear how immersive VR compares to mobile games for learning (Wu and Lee, 2015) and the portable nature of mobile communication may facilitate a different learning potential about science. Therefore, a fruitful line of research we intend to conduct will investigate several teaching methods to isolate the technologies that best support student learning about complex social and scientific issues.

Our student and public samples are admittedly not representative of broader populations. Future work should therefore attempt to expand the participant base and extend the findings to additional communities. Our immersive VR treatment was also shorter than many types of educational curricula. As immersive VR becomes more user-friendly and common, the length or types of curricula can expand.

Finally, our experiments lacked a true control conditions to compare against the immersive VR treatments. To more thoroughly evaluate the effect of immersive VR on learning about climate change, immersive VR should be compared to other (non-immersive) media and different learning experiences to evaluate learning gain potential.

The future of using immersive VR for education is promising, though we do not expect the technology to replace teachers, just like Massive Open Online Courses have not replaced universities or in-person lectures. However, immersive VR has the potential to provide unique opportunities for education and to reach students with special learning needs. As such, we strategically positioned the studies and experiences in this paper as field trips (Borst et al., 2018). Students do not participate in field trips every day, but only on special occasions and for more hands-on, experiential learning. We therefore can see the immediate potential of immersive VR for tutoring and classrooms that need topical reinforcement. For example, students struggling in chemistry can go deeper into the material and learn about the composition of elements first-hand in immersive VR. We currently view immersive VR as a medium to supplement the conventional learning environment, not replace it.

## Ethics statement

This study was carried out in accordance with the recommendations of the Stanford University Institutional Review Board with written informed consent from all subjects. All subjects gave written informed consent in accordance with the Declaration of Helsinki. The protocol was approved by the Stanford University Institutional Review Board.

## Author contributions

DM wrote the paper and reanalyzed the data. RL collected data for Studies 3–4, edited the paper, and analyzed the original data. BP collected data for Studies 1–2, edited the paper, and assisted with study design. RP edited the paper, assisted with study design, and oversaw study execution. JB edited the paper, assisted with study design, and oversaw study execution.

### Conflict of interest statement

The authors declare that the research was conducted in the absence of any commercial or financial relationships that could be construed as a potential conflict of interest.
