# Supplementary material for: Immersive Virtual Reality Field Trips Facilitate Learning About Climate Change
Source: Front Psychol. 2018 Nov 30;9:2364. doi: 10.3389/fpsyg.2018.02364 (PMC6284182; doi:10.3389/fpsyg.2018.02364)
Supplement: Supplementary file 1 [file Table_1.DOCX]

**Appendix A: Ideal Responses to Ocean Acidification Questions**

Questions were graded on a 0, 1, 2-point scale.

*0 points*: no answer, “I don’t know”, a simple restatement of the question (“ocean acidification is acid in the ocean”), or an entirely or almost entirely incorrect answer.

*1 point:* a partially correct answer that includes some portions of the ideal answer but is either missing pieces or has additional incorrect information added (“ocean acidification is a buildup of carbonic acid in the oceans from volcanic activity”); for numerical answers, an answer that is within approximately 50% of the correct answer.

*2 points:* an answer that contains all or almost all of the elements of the ideal answers, with only minor omissions or inaccuracies (“ocean acidification is a build-up of acid from CO2 in the oceans”); for numerical answers, an answer that is within 10% of the correct answer.

Ideal Responses (questions are in **bold**)

1. **What is ocean acidification?** It is the build-up of carbonic acid in the ocean as a result of increasing levels of atmospheric carbon dioxide.
2. **What is the most significant cause of ocean acidification?** Human-produced atmospheric carbon dioxide from sources such as burning fossil fuels.
3. **What are some of the predicted effects of increasing ocean acidification on the world’s ocean ecosystems? Be as detailed as you can (need at least 2 correct effects for a 2-point answer).** Many species will die off or experience population decline, but not all. Ocean species with hard exoskeletons, such as coral and sea snails, will have trouble building and maintaining their exoskeletons. Others, such as sea urchins, will produce smaller larvae and experience acidosis, a build-up of carbonic acid in their bodies, slowing their metabolisms. Some species of fish will die off due to the loss of food sources. Other species that are more tolerant of the acidity will move in and displace the weakened species.
4. **How do coral and other sea creatures build their hard skeletons and shells?**They collect Calcium (Ca2+) + and bicarbonate (2HCO3-) ions from the water around them and combine them to make calcium carbonate (CaCO3), the main component of their skeletons.
5. **How much carbon dioxide is absorbed by the planet’s oceans each year?**

Two billion tons

OR

25% of the carbon dioxide emitted by human activity.

1. **How much has the pH level of the ocean changed since the industrial revolution?**

One tenth of a pH unit

OR

a 30% increase in acidity

**Appendix B: Change in Pre- and Post-Test Measures Correlations (Study 2)**

|  | OAK_Change | CNS_Change | NEP_Change | Presence |
| --- | --- | --- | --- | --- |
| OAK_Change | -- | .26^†^ | .21 | .28^†^ |
| CNS_Change | .26^†^ | -- | .10 | .33* |
| NEP_Change | .21* | .10 | -- | .32* |
| Presence | .28^†^ | .33* | .32* | -- |

*Note*. ^†^ *p* < .08, * *p* < .05

**Appendix C: Study 3 Head and Hand Movement Descriptives**

**Head movements.** Once every second, we recorded the total movement of the participant’s head in six degrees of freedom. The head translations were recorded in meters (m) along three degrees of freedom (*M* = 26.01 m; *SD* = 7.45 m) and head rotations were recorded in degrees (°) along three degrees of freedom (*M* = 10,718.55°; *SD* = 2,800.75°).

**Hand movements**. We further recorded the total physical movement of the participant’s right and left hands, each in six degrees of freedom. The translations were recorded in meters (m) along three degrees of freedom for each of the right (*M* = 79.49 m; *SD* = 18.11 m) and the left hands (*M* = 76.09 m; *SD* = 17.49 m). Rotations were recorded in degrees along three degrees of freedom for the right (*M* = 18,277.62°, *SD* = 4,856.50°) and the left hands (*M* = 18,381.42°, *SD* = 4,866.60°).

**Appendix D: Simulator Sickness Questions and Temperature Estimation**

**Simulator Sickness**

We included simulator sickness to explore if the experimental conditions affected participants after the immersive VR experience. The measure in the post-questionnaire included three questions on a five-point Likert scale, soliciting how much general discomfort, eyestrain, and nausea occurred (Cronbach’s α = 0.65; Kennedy, Lane, Berbaum, & Lilienthal, 1993).

All responses were recorded on a five-point scale, ranging from “Not at all” to “A great deal:”

1. General discomfort
2. Eyestrain
3. Nausea

**Temperature**

We recorded participants’ estimates of room temperatures before (*M* = 69.07 °F; *SD* = 3.15 °F) and after (*M* = 69.51 °F; *SD* = 3.57 °F) the VR experience. Participants also self-reported estimates of relative changes (Δ) in temperature since the last time they reported, on a five-point scale ranging from *a lot colder* to *a lot warmer*. Participants perceived steady increases in temperature after each stage, including after completing the VR experience in the first zone (M = 2.09 °FΔ; SD = 0.81 °FΔ), in the second zone (M = 2.09 °FΔ; SD = 0.78 °FΔ), and in the third zone (M = 2.16 °FΔ; SD = 0.87 °FΔ).

**Appendix E: Ideal Responses to Ocean Acidification Questions for Study 4**

Questions were graded on a 0, 1, 2-point scale.

*0 points:* no answer, “I don’t know”, a simple restatement of the question (“ocean acidification is acid in the ocean”), or an entirely or almost entirely incorrect answer.

*1 point:* a partially correct answer includes some portions of the ideal answer but is either missing pieces or has additional incorrect information added (“ocean acidification is a build-up of carbonic acid in the oceans from volcanic activity”). For numerical answers, an answer that is on the same order of magnitude as the correct answer is acceptable for 1 point.

*2 points:* an answer that contains all or almost all of the elements of the ideal answers, with only minor omissions or inaccuracies (“ocean acidification is a build-up of acid from CO2 in the oceans”). For numerical answers, an answer that is within 10% of the correct answer is acceptable for 2 points.

Ideal Responses (questions are in **bold**)

1. **What is ocean acidification?** Ocean acidification is the build-up of carbonic acid in the ocean as a result of increasing levels of atmospheric carbon dioxide.
2. **What are the causes of ocean acidification?** Human-produced atmospheric carbon dioxide from sources such as burning fossil fuels.
3. **What are the predicted effects of ocean acidification?** Many species will die off or experience population decline, but not all. Ocean species with hard exoskeletons, such as coral and sea snails, will have trouble building and maintaining their exoskeletons. Others, such as sea urchins, will produce smaller larvae and experience acidosis, a build-up of carbonic acid in their bodies, slowing their metabolisms. Some species of fish will die off due to the loss of food sources. Other species that are more tolerant of the acidity will move in and displace the weakened species.

**Additional Correlations for Study 4**

Average attitude toward ocean acidification was positively correlated with the CNS (*ρ* = 0.53, *p* < .001) and NEP scales (*ρ* = 0.62, *p* < .001). Pre- (*ρ* = 0.36, *p* = .017) and post-test (*ρ* = 0.46, *p* = .002) levels of ocean acidification knowledge were positively correlated with the NEP scale.
